# Live cell X-ray imaging of autophagic vacuoles formation and chromatin dynamics in fission yeast

**DOI:** 10.1038/s41598-017-13175-9

**Published:** 2017-10-23

**Authors:** Natalja Strelnikova, Nora Sauter, Manuel Guizar-Sicairos, Michael Göllner, Ana Diaz, Petrina Delivani, Mariola Chacón, Iva M. Tolić, Vasily Zaburdaev, Thomas Pfohl

**Affiliations:** 1 Department of Chemistry, University of Basel, Basel, Switzerland; 2 Paul Scherrer Institut, Villigen, Switzerland; 3 Max Planck Institute of Molecular Cell Biology and Genetics, Dresden, Germany; 4 Division of Molecular Biology, Ruđer Bošković Institute, Zagreb, Croatia; 5 Max Planck Institute for the Physics of Complex Systems, Dresden, Germany; 6 Biomaterials Science Center, University of Basel, Basel, Switzerland; 7 Present Address: Institute of Physics, University of Freiburg, Freiburg, Germany

## Abstract

Seeing physiological processes at the nanoscale in living organisms without labeling is an ultimate goal in life sciences. Using X-ray ptychography, we explored *in situ* the dynamics of unstained, living fission yeast *Schizosaccharomyces pombe* cells in natural, aqueous environment at the nanoscale. In contrast to previous X-ray imaging studies on biological matter, in this work the eukaryotic cells were alive even after several ptychographic X-ray scans, which allowed us to visualize the chromatin motion as well as the autophagic cell death induced by the ionizing radiation. The accumulated radiation of the sequential scans allowed for the determination of a characteristic dose of autophagic vacuole formation and the lethal dose for fission yeast. The presented results demonstrate a practical method that opens another way of looking at living biological specimens and processes in a time-resolved label-free setting.

## Introduction

Studies of nanoscale structures and dynamics of biological matter greatly benefit from observing samples in living state using label-free methods^1^. X-ray ptychography enables quantitative visualization of whole biological cells with nanoscale resolution based on the natural electron density contrast of the cell content^2–5^. An ideal eukaryotic model organism for cellular dynamic studies is fission yeast at the horsetail stage owing to the oscillations of meiotic chromosomes in the time scale of minutes to hours^6–8^. Moreover, intracellular structure changes caused by X-ray radiation are of interest for a direct analysis *in situ* ^9^. The major challenge of X-ray imaging of living cellular specimens is the very low lethal radiation dose, and owing to the intense radiation damage and a low electron density contrast, sequential X-ray imaging of live eukaryotic cells was not possible so far^10–13^. To record sufficient information, the samples have to be exposed to a certain amount of radiation, which has to be greater than the minimum of the required dose for imaging and less than the maximum tolerable dose for the specimen^10^. The limiting factor of the resolution is therefore set by the X-ray radiation dose^10,11^. Natural, aqueous environments of biological specimens significantly decrease the electron density contrast, thus higher X-ray flux is required, which consequently increases the radiation dose needed for a given resolution^10^. Radiation induced degradation can be reduced by chemical or cryo-fixation. Using cryo-fixation, X-ray ptychography^2,4,14^ and diffraction microscopy^15,16^ of frozen hydrated cells were accomplished. More advanced X-ray studies were realized on living cells^17,18^ and appear to be a promising approach to analyze cellular processes *in situ*. Imaging of initially alive cells simplifies sample preparation, owing to the fact that fixation steps are not required. Imaged living cells died during the first X-ray scan^18^ or after a free electron laser (FEL) pulse^13^, due to the lethal radiation dose. However, first electron density maps measured with X-rays of initially alive bacteria, which were obtained with less than a lethal dose, were recently presented^12,19^. X-ray ptychography is a coherent diffractive imaging (CDI) technique that combines scanning microscopy with advanced phase retrieval algorithms^20–22^. It relies on scanning the extended sample by the X-ray beam, collecting 2D diffraction patterns from a number of overlapping regions of the specimen and a subsequent iterative reconstruction of a single projection image, which is consistent with all recorded diffraction patterns^21^.

## Results

### X-ray ptychography

**Figure 1:**
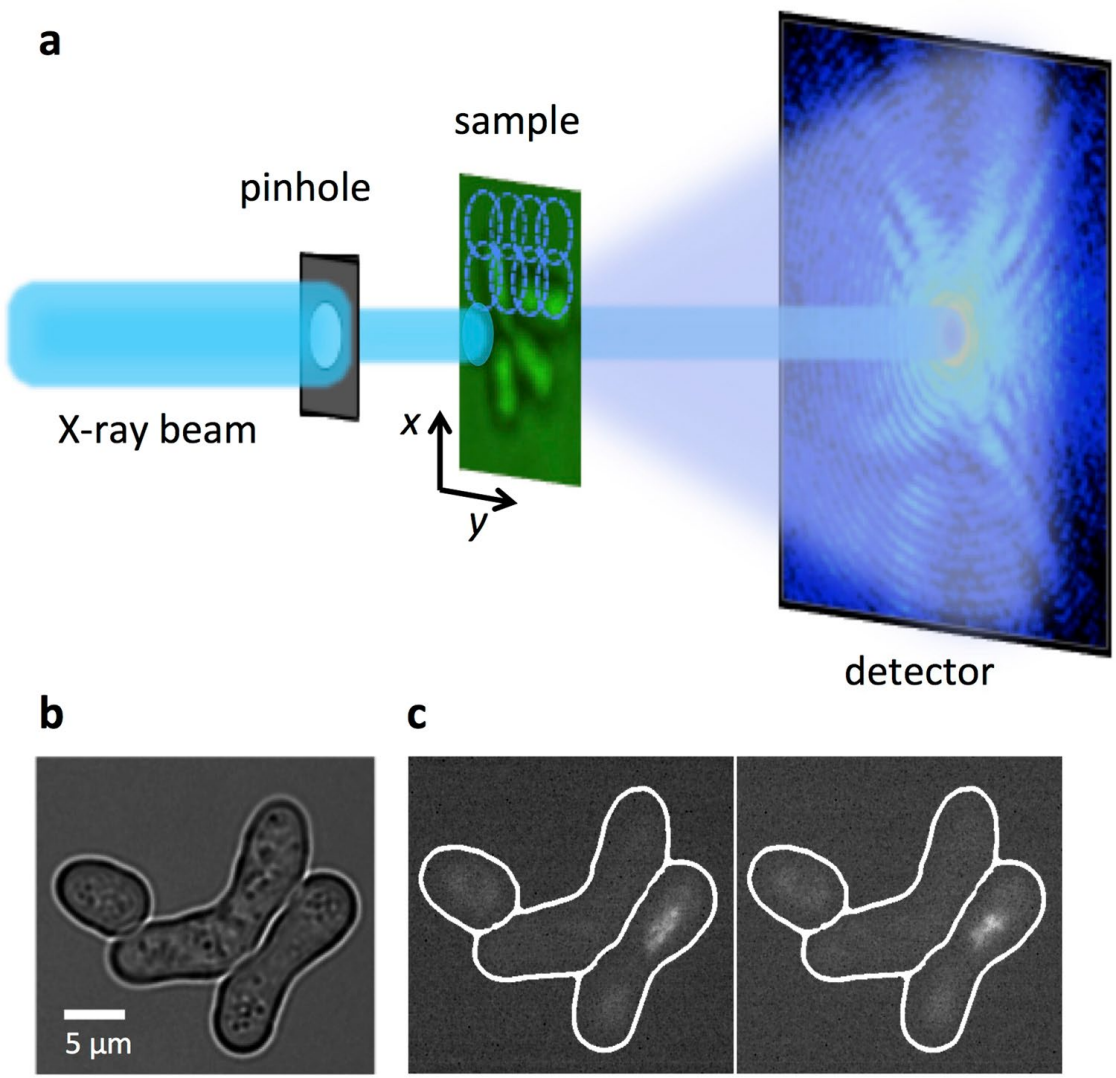
Schematic representation of the experimental ptychography setup for imaging living cells. (**a**) For the X-ray ptychography experiments, a monochromatic (*λ* = 0.2 nm) beam was used to coherently illuminate a pinhole. The cell sample was scanned to collect a series of diffraction patterns from partially overlapping illuminated regions, which allow for a robust image reconstruction. The high dynamic range and count rate of the detector allows us to record the full dynamic range of the 2D diffraction patterns at the detector and avoid a loss of low spatial-frequency information that would occur if a beamstop was used. (**b**) A visible light bright-field optical micrograph shows three fission *Schizosaccharomyces pombe* yeast cells under nitrogen starvation conditions, where two of them were banana-shaped zygotes. (**c**) Corresponding fluorescence microscopy images of the same cells as in (**b**) in a time interval of 5 min are shown. In order to distinguish zygotes with moving chromosomes, ‘nuclear oscillations’, among cells with ‘non-oscillating’ ones, the rec25 gene was labeled with green fluorescent protein (GFP) and used as an indirect marker of DNA double strand breaks^24^. Here, only one of two zygotes was at the horsetail stage.

Ptychography is an X-ray imaging technique with spatial resolution limited in principle by the spatial wavelength of the incident beam and the maximum angle at which diffracted signal can be measured with sufficient signal-to-noise ratio, although in practice the resolution can be also limited by scanning precision or radiation induced damage on the specimen under study (Fig. 1). In living samples, intracellular motions are happening during a single scan which can blur the images. A ptychography setup with a pinhole-defined illumination was chosen in order to achieve good contrast and high resolution with a reduced dosage of radiation (Fig. 1A) due to the broad spatial spectrum^23^. We obtained reconstructed images with pixel sizes of 45 × 45 nm^2^ and an estimated resolution in the range of 100–200 nm of live cells in aqueous environment and reduced the radiation doses down to about 10^3^–10^4^ Gy per scan. These doses are two to three orders of magnitude less than 1.8 × 10^6^ Gy recorded for recent X-ray images of (initially) alive eukaryotic cells^18^ and close to the dose of 8.9 × 10^3^ Gy used for holographic imaging of living bacteria^19^.

Here, we explore the dynamics of living fission yeast *Schizosaccharomyces pombe* cells during meiosis in natural, aqueous environment *in situ*. Fission yeast cells are ideal eukaryotic model organisms, because many of basic cellular principles and cell regulators are conserved from yeast to humans^8^. Meiosis in fission yeast is induced by depleting nitrogen sources from the culture medium and haploid cells of the opposite mating type conjugate and form a diploid “banana-shaped” zygote^6^ (Fig. 1b,c). At the horsetail stage of meiosis strong oscillations of chromosomes can be observed with extended periods of chromosomal back and forth motions along the cell axis^25^. The period of an individual oscillation is about 10–15 min^6^. After several hours, at the end of the horsetail stage, the oscillations slow down and finally stop.

### X-ray induced autophagy in fission yeast cells

**Figure 2:**
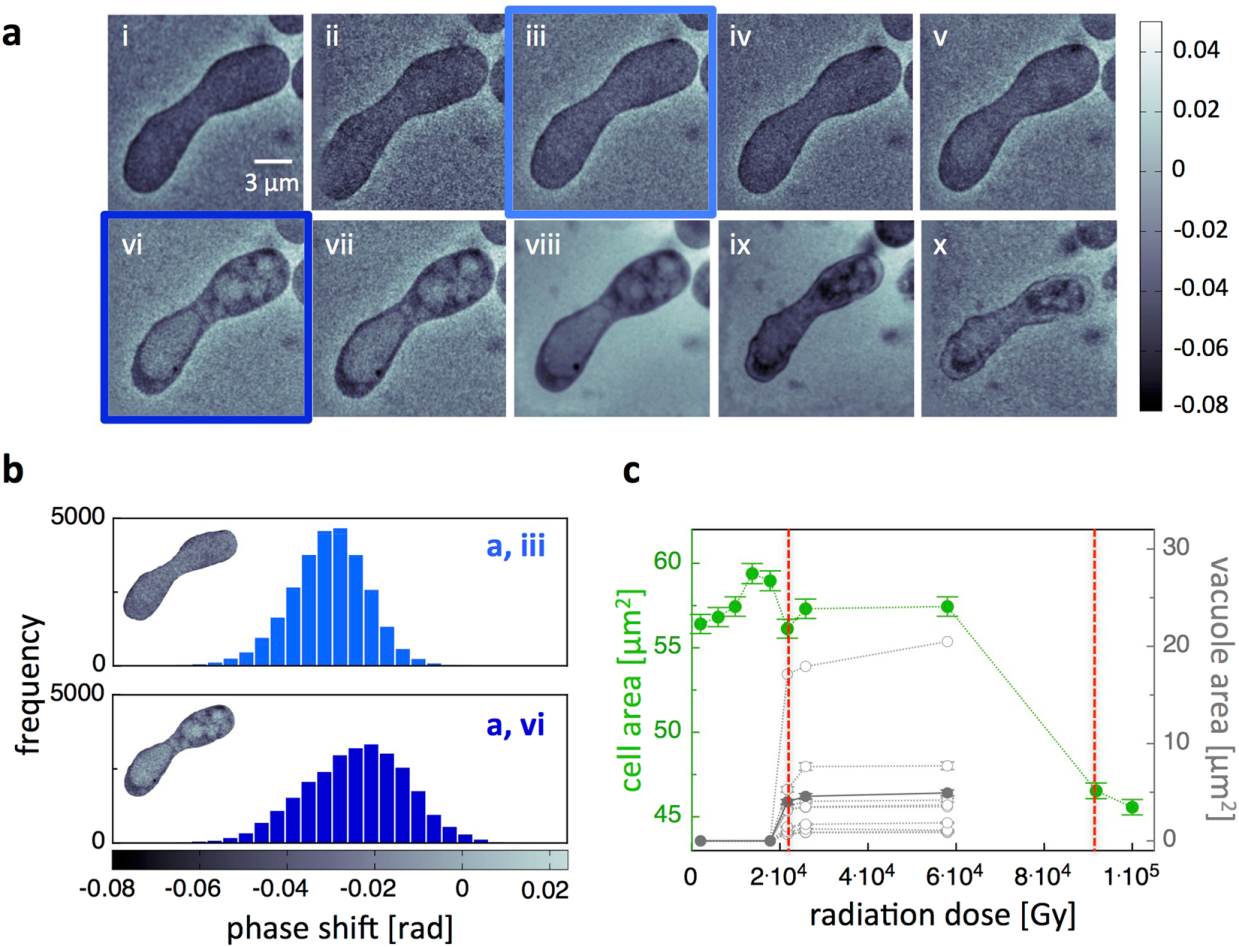
X-ray ptychography images of a live fission yeast zygote. (**a**) Successive image sequence of a live fission yeast zygote obtained by ptychographic CDI scans. (**b**) Phase shift histograms (plot of the number of pixels with a specific phase shift) of a zygote in the image without autophagic vacuoles (a, iii) and with autophagic vacuoles (a, vi). A positive shift corresponds to a lighter color. (**c**) Projected cell area and projected vacuole area versus the radiation dose calculated from the ptychographic zygote images. Each set of grey circles connected by a line corresponds to a different vacuole and the filled circles correspond to the mean area of the vacuoles. A characteristic dose of the onset of vacuole formation and a lethal dose can be identified (red dashed lines).

X-ray ptychography micrographs of a successive image sequence of a fission yeast zygote and an analysis of the impact of ionizing radiation on the cell are shown in Fig. 2. During the first four scans, no structural changes - almost homogenous density within the entire cell - of a zygote were observed. A further exposure of X-rays in the successive scans led to the appearance of clear, light and rounded structures in the zygote. These observed cellular structures may be a signature of a radiation induced formation of vacuoles^26^ and autophagic bodies^27^, which were described for fission yeast cells and might be a visual indication of autophagy^28–30^. These structural changes coincided with an overall positive shift in the phase shift histograms of zygote images showing autophagic vacuoles in comparison to zygote images without vacuoles (Fig. 2b). Increasing the radiation dose further, a bursting of the membrane and shrinkage of the cell was observed (Fig. 2a **-ix,x**), which demonstrates that after accumulating a certain amount of radiation the zygote perished. To characterize the dynamics of the autophagic vacuole formation and cell lysis, changes of the projected zygote area and of the projected area of individual vacuoles were analyzed (Fig. 2c). In the first four ptychography scans, a slight increase of the projected area of the zygote was observed. At an accumulated dose of about 2.2 × 10^4^ Gy (sixth scan) for the particular scan shown in Fig. 2c, the area decreased back to its initial size and the autophagic vacuole formation set in, a first critical dose for live fission yeast cells can be defined. Applying more X-ray radiation, the initial number of vacuoles did not change, whereas their volume (projected area) increased until the cell burst. At a dose of about 9.2 × 10^4^ Gy for this particular cell, the bursting of the cell membrane was concurred by a strong decrease of the projected zygote area. The observed formation of vacuoles can be attributed to ionizing effects of the X-ray radiation, which induce radiolysis of water and cause hydrogen peroxide (H_2_O_2_) and hydroxyl radical (OH^.^) formation^27^. These very reactive oxygen species (ROS) activate protein kinases in yeast^9^ and can cause autophagy^31^. Apart from the fact that the zygote was exposed to X-rays, yeast cells grew under nitrogen depletion, which might be a stimulus to facilitate autophagy if cells starve for several hours^32^. However, the autophagic vacuole formation was observed only after several ptychography scans, and was not observed in comparable experiments using optical microscopy.

In total, nine individual live fission yeast zygotes were analyzed. The average radiation dose at which autophagic vacuole formation occurred was about (3.30 ± 0.74) × 10^4^ Gy. When the radiation dosages accumulated to (9.6 ± 3.2) × 10^4^ Gy, zygotes were lysed, which can be defined as the lethal radiation dose for fission yeast. Interestingly, non-meiotic cells were more resistant to X-ray radiation, where vacuole formation occurred at higher doses of (8.7 ± 3.8) × 10^4^ Gy and cell death at about (1.20 ± 0.19) × 10^5^ Gy, indicating that the lethal radiation dose of non-meiotic cells is similar to zygotes, whereas the vacuole formation in non-meiotic cells occurs at much higher radiation doses.

### X-ray imaging of the chromosome motion

**Figure 3:**
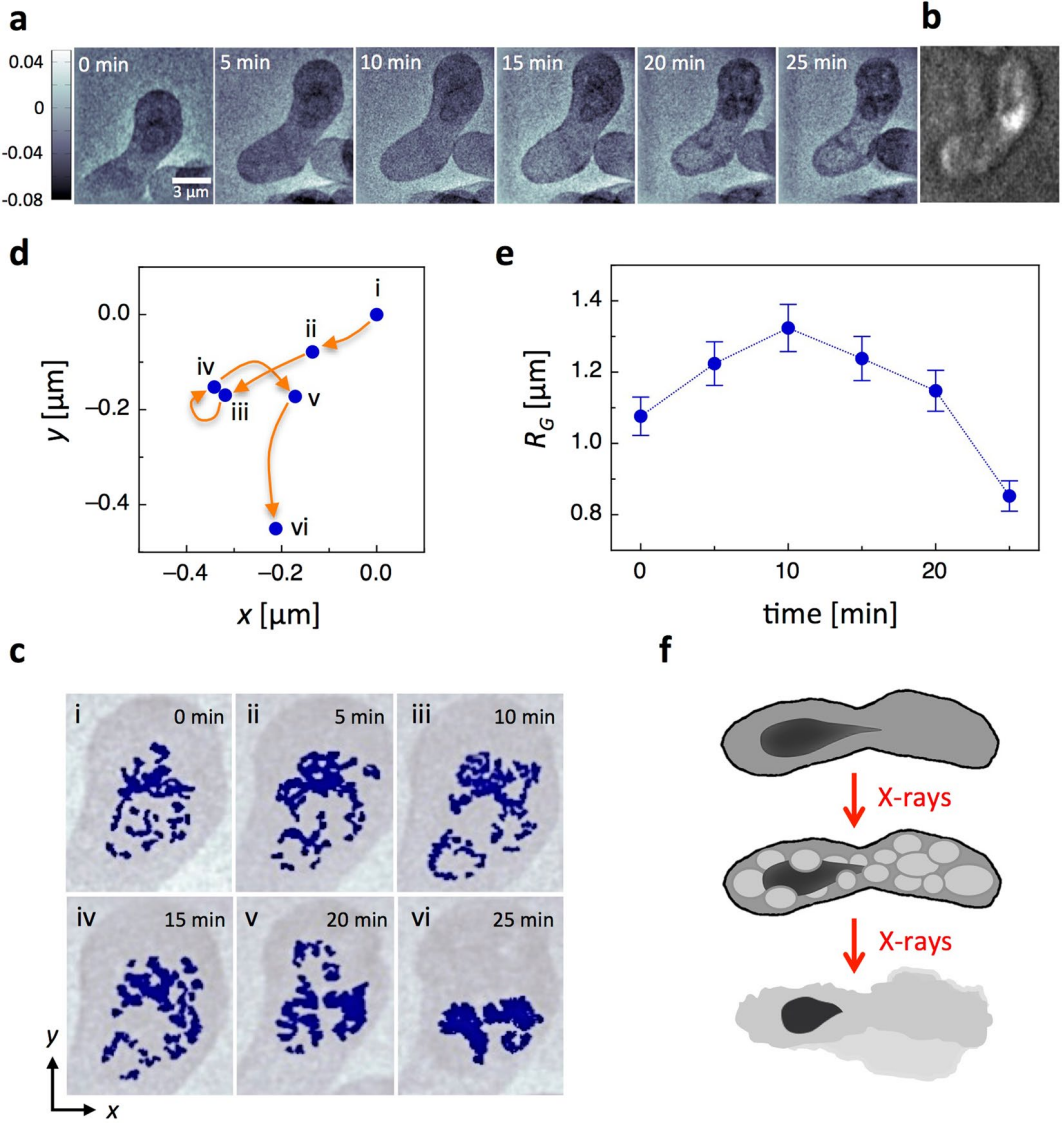
X-ray ptychography images of chromosomes motion in a live fission yeast zygote at the horsetail stage. (**a**) Temporal image sequence of a live zygote in the horsetail stage obtained by X-ray ptychography. (**b**) Fluorescence micrograph of the zygote taken before the X-ray ptychography scans. (**c**) Overlay of image processed contours of the chromosomes (dark blue) on the original ptychography images. (**d**) Center of mass motion of chromosomes between the sequential ptychographic images. The *x* and *y*-position of the center of mass of the first scan at 0 min was set to 0,0. (**e**) Calculated radius of gyration of the chromosomes versus time. (**f**) Schematic representation of autophagic vacuoles formation and cell lysis during meiotic chromosome oscillations.

To further demonstrate the potential of ptychography for studying cellular dynamics, we imaged meiotic yeast zygote at the horsetail stage. During this stage, an extended movement of the whole chromatin happens, and at the same time, the motion matches the time scales of the X-ray imaging technique. Freshly prepared samples were firstly analyzed with a fluorescence microscope to find a zygote at the horsetail stage in the sample and then mounted on the X-ray ptychography setup. A temporal sequence of six X-ray ptychography images of a live fission yeast zygote in the horsetail stage is presented in Fig. 3a. Interestingly, a darker (a more negative phase shift) and denser structure in the top part of the zygote was observed. This structure was moving during one scan to another and furthermore changed its shape. We identified this densified structure as moving chromatin, which has the same appearance as the chromosomes in the fluorescence micrograph taken before the X-ray ptychography scans (Fig. 3b). Image processed contours of the chromosomes overlaid on the original ptychography images are shown in Fig. 3c. The motion of the chromosomes was analyzed by calculating their center of mass. Starting from the initial position of the chromosomes the subsequent center of mass positions showed a movement of several hundreds of nanometer away from the upper cell end in the direction of the lower part of the cell (Fig. 3d). For further analysis, we compared the shape changes of the chromosomes over time by calculating the radius of gyration ${R}_{{\rm{G}}}=\sqrt{\frac{1}{N}\sum _{i=1}^{N}{({\overrightarrow{r}}_{i}-{\overrightarrow{r}}_{{\rm{C}}{\rm{M}}})}^{2}}$, where $N$ is the number of pixels of the chromatin, $\vec{{r}_{i}}$ are the position vectors and ${\overrightarrow{r}}_{{\rm{C}}{\rm{M}}}$ is the center of mass of the chromosomes. The radius of gyration, which characterizes the packing and shape of the chromosomes, versus time is plotted in Fig. 3f. Firstly, a looser packing of the chromosomes, bigger *R*_G__g_, was observed, reaching a maximum of *R*_G_ = (1.32 ± 0.07) µm after 10 min during their motion to the lower cell end. Afterwards, *R*_G_ steadily decreased to a minimum of (0.85 ± 0.04) µm, which characterizes a strong compaction of the chromosomes. This chromosome compaction was found in conjunction with the formation of autophagic vacuoles, which could be observed after about 20 min (Fig. 3a). The motion of the chromosomes was slowed down in comparison to the observed period of the oscillation of about 10–15 min^6^. This observed deceleration of the chromosome motion is an indication of an impact of X-ray radiation, which most probably damages the cell at the molecular level; especially the appearance of autophagic vacuoles in concurrence with the chromosomes compaction had a strong impact on the oscillating chromosomes. Moreover, due to a time-consuming sample preparation and mounting procedure, the X-ray ptychography images were presumably taken at the end of the horsetail period, when the oscillations slowed down. The appearance of autophagic vacuoles was observed, when the radiation dose accumulated to about 1.2 × 10^4^ Gy. This dose of the vacuole formation is less than half of the dose, which was found for normal yeast zygotes, which might be an indication that zygotes in the horsetail stage are more sensitive to X-ray radiation.

## Conclusions

In these experiments, we optimized the sample preparation and experimental setup to successfully apply ptychography for sequential imaging – producing X-ray movies – of live meiotic yeast cells in aqueous environment and established a method to investigate intracellular nanostructures. Based on the natural electron density contrast, this label-free imaging method allowed us to visualize cellular structures *in situ*. We discovered autophagic cell death or type II programmed cell death^28^ and cell lysis, induced by the pathological environment due to the ionizing X-ray radiation. Autophagy in the yeast and mammalian cells is similar^30^ and it is considered to play an important part in the response of radiation therapy^33^. Radiation-induced  damage on the molecular level most probably occurred already during the first X-ray scan, but it did not cause visible cell changes and the cell stayed alive. Thus an average radiation dose at which visible signs of autophagy occurs by formation of autophagic vacuoles^32^ and a characteristic dose of membrane bursting of the eukaryotic cells can be obtained. Moreover, the dynamics of denser structures, which are most likely chromatin structures at the end of their oscillatory motion during the horsetail stage of yeast zygotes, can be imaged and analyzed. However, zygotes are most likely already damaged at the molecular level by radiation. This imaging approach also simplifies sample preparation and avoids artifact formation caused by fixation, sectioning or labeling.

We believe that improved sample environments, e.g. microfluidics setup^34^ to flush fresh medium and remove free radicals^35^, modified scanning protocols and adjusted ranges of interest will further advance, as here demonstrated, the way of seeing physiological processes of individual eukaryotic cells as well as tissues with subcellular, nanoscale resolution.

## Methods

In order to avoid a strong background signal and to create a cytocompatible environment, we used biocompatible and X-ray resistant 200 nm thick silicon nitride (Si_3_N_4_) membrane windows (frame: 5 × 5 mm^2^ × 200 µm, membrane: 1.5 × 1.5 mm^2^ × 200 nm; Silson Ltd, Blisworth, England)^17,18,34^. The Si_3_N_4_ membrane windows were coated with lectin (Sigma-Aldrich, St. Louis, MO, USA) to increase cell adhesion to the membrane surface, which is crucial for the spatial stability of the cells and thus for the reproducibility of X-ray ptychography scans. Fission yeast *Schizosaccharomyces pombe* cells were kept in phosphate-buffered saline (PBS) medium at room temperature. For fluorescence optical imaging, we used the genotype of the fission yeast strain h90 rec25::GFP-KanMX6. The strain was a kind gift from C. Martín-Castellanos (CM62, IBFG, Salamanca, Spain). To induce meiosis, fission yeast cells were transferred to an Eppendorf tube with 100 µl of nutrition deficient Edinburgh minimal medium (EMM-N)^7^ and kept for 30 min at room temperature. Afterwards, a small droplet of medium with cells were put on a lectin-coated Si_3_N_4_ membrane window and the device was covered by an uncoated Si_3_N_4_ membrane window and accurately glued with UHU plus epoxy quick set adhesive at the edges of the membranes. The described procedure enables the preparation of hydrated living cell samples for X-ray experiments with an intercalated aqueous film of 5 to 10 µm in thickness. The gap was determined based on bright field optical imaging where the distance between the membranes was comparable to the size of the cell as observed by changing the focus. Since the aqueous environment drastically decreases the electron density contrast of the sample, a small sample thickness is crucial to reduce the background signal caused by the medium in the device.

X-ray ptychography experiments were performed at the coherent small-angle X-ray scattering (cSAXS) beamline of the Swiss Light Source, Paul Scherrer Institut, PSI, Villigen, Switzerland. The schematic representation of the setup is shown in Fig. 1a. An X-ray beam of 6.2 keV photon energy, *λ* = 0.2 nm, was selected using a double crystal Si (111) monochromator. The incident beam was defined by a pinhole with a transverse diameter of about 2.5 µm in order to obtain a coherent spatially confined illumination at the sample, which was placed 3 mm downstream of the pinhole and had at the sample position approximately the same diameter. The sample of hydrated live cells was placed on a piezoelectric scanning stage to allow for nanometer precision scanning. The coherent X-ray beam diffracted by the sample propagates through a helium flushed flight tube to a photon-counting Pilatus 2D detector^36^, which is located at distance of 7.412 m from the sample. The broad angular spectrum of a pinhole-defined illumination is well suited for minimizing the radiation dose while acquiring images with good contrast and a moderately high resolution^23^. Before the X-ray ptychography experiments, the cell samples were imaged by fluorescent microscopy in order to identify oscillating zygotes. The membranes were then mounted on the setup and the identified cells were positioned using an on-stage bright field microscope.

In order to avoid the raster grid pathology^37^ all scans were performed following a Fermat spiral scanning pattern^38^. In order to find optimal scanning parameters, different step sizes and exposure times were applied. For the measurements in Fig. 2a a scanning field of view of 18 × 14 µm^2^ and an average step size of 0.7 µm were used with an exposure time of 0.1 s per scanning point. For these parameters the resolution was about 200 nm with an average flux of about 7.4 × 10^5^ photons/µm^2^. To calculate the average flux we first normalized the reconstructed illumination intensity using the total number of counts arriving at the detector after compensating for absorbing and scattering elements in the path of the beam, then we used the scanning pattern to generate a grid of the distribution of photons incident on the sample for the whole scan. The flux in photons/µm^2^ is finally calculated by integrating over an area significantly larger than the illumination and dividing by the area, in this manner we included in the calculation the total dose incident on the sample including the overlapping regions of the scan^39^. The corresponding dose of 3.9 × 10^3^ Gy was estimated as described in ref.^16^: The dose, *D*, is calculated based on the surface dose equation ${D}=\mu {N}_{0}h\nu /\rho$ ^10^, where the attenuation length, 1/*μ*, was obtained from tabulated values^40^ assuming an average composition of H_128_C_30_N_9_O_49_S_1_ and average density of *ρ* = 1 g cm^−3^.

In order to observe the cell behavior (death or ability to recover) between the X-ray scans, the time interval among the images was different: 5 min, 25 min, 20 min, 20 min, 50 min, 30 min, 40 min, 2 h, 3 h. This allows us to assume that the cell death is initiated by the X-ray radiation and does not occur/continue when the X-rays are switched off. The radiation doses used for single ptychography scans in Fig. 2a were different, the 1^st^ scan 1.96 × 10^3^ Gy, 2^nd^ scan 4.01 × 10^3^ Gy, 3^rd^ scan 3.87 × 10^3^ Gy, 4^th^ scan 3.86 × 10^3^ Gy, 5^th^ scan 4.09 × 10^3^ Gy, 6^th^ scan 3.87 × 10^3^ Gy, 7^th^ scan 4.14 × 10^3^ Gy, and 10^th^ scan 8.20 × 10^3^ Gy. The quality of the images in Fig. 2a(viii, ix) and resolution down to about 100 nm was improved using smaller step sizes of 0.5 µm and longer exposure times of 0.4 s, which increased the radiation dose to about 3.3 × 10^4^ Gy.

The images in Fig. 3A were obtained with an exposure time of 0.1 s per scanning point and an average step size of 1 µm. The flux at the sample position was 4.2 × 10^5^ photons/µm^2^ for the individual scans, corresponding to an estimated resolution of about 100 nm. The radiation doses per scan were 1.93 × 10^3^ Gy (1^st^), 1.83 × 10^3^ Gy (2^nd^), 2.71 × 10^3^ Gy (3^rd^), 2.70 × 10^3^ Gy (4^th^), 2.76 × 10^3^ Gy (5^th^), and 2.74 × 10^3^ Gy (6^th^).

Reconstructions were carried out using the maximum likelihood method through non-linear optimization^20,22^. In order to reduce the noise in the reconstructions, gradient preconditioning and regularization, as described by Thibault and Guizar-Sicairos^22^, were used. A good estimate of the incident illumination is important to facilitate the reconstruction of weak contrast specimens^41^, such as the hydrated live cells presented here. For this purpose, we characterized the incident illumination via ptychography before the experiments using a 2D test patterns similar to those used in^42^. The illumination phase and amplitude profile were stable for the duration of a single scan. For this case, the resolution of the reconstruction could not be assessed via Fourier shell correlation (FSC)^43,44^, because two identical datasets were not available due to changes or movement of the live specimens. To assess the resolution of each image we used instead a method based on the angular-averaged power spectral density (PSD) method as described in^14^.

X-ray ptychography images were analyzed using ImageJ (version 1.47k, Wayne Rasband, National Institute of Health, USA) and MATLAB (version R2012b, The MathWorks, Natick, USA) by applying custom developed scripts. The images were first denoised by conditional mean filtering resulting in an edge preserving smoothing. Further applying edge detection algorithms yield the contour of the cells, which acts as the range of interest in order to find the chromosomes by local thresholding.
